# West Chicago Medical Society

**Published:** 1881-07

**Authors:** 


### ARTICLE VII.

### WEST CHICAGO MEDICAL SOCIETY.

Dr. H. M. Lyman, President, read a paper on the

### IDENTITY OF SMALL-POX AND COW-POX.

Soon after Jenner's discovery, a Mr. Ceely of England, experimented in communicating small-pox to cows, and after a few re-vaccinations, obtained common vaccine virus. It seems these experiments were ignored by most physicians of the present day. However, the late investigations of Pasteur with chicken cholera threw more light on the subject. Experiments similar to Ceely's had been made in Berlin in 1802. It was remarkable that cowpox had become so rare since those days. It was not a primary disease in cows, and must have originated from variolous milkers' hands. No disease had so many forms with different degrees of intensity as variola.

The present theory was the only one which could explain the immunity conferred by vaccinia, for a disease never protected from another disease. Thus anthrax vaccination could be accounted for as well. And in cases of emergency, he advocated originating new vaccine virus by inoculating cows with small-pox. The first vaccine thus obtained would almost have the character of variola, and should be first inoculated in another cow to investigate its intensity. It would be more likely to produce vaccinia with an eruption.

In Jenner's time, vaccination was thought to be a protective for life, and the statistics furnished very few cases of small-pox after vaccination, and obviously during the following seven years. That vaccine virus had partly lost its efficacy in England was a fact, and a time could be calculated when it would become quite innocuous if not renewed. The virus used in this country is of a more recent origin. The mortality from small-pox was less before Jenner's time than it is now. For through a series of ages small-pox, by perpetuating itself in man, had become partly attenuated (the fittest having survived), those whose systems had been least able to resist having died, and a sort of tolerance had been conferred through hereditary descent.

It was probable that variola had a *habitat* in China, or Central Asia, whence most epidemics seemed to have arisen. According to this theory, epidemics would stop after many had had the disease, and many others had obtained a sort of immunity from it through exposure to the epidemic influence. Dr. Lyman's paper is printed in full in this number of the JOURNAL.

DISCUSSION.

Dr. Norman Bridge, referred to Dr. Meyer's experiment in this country, by which an epidemic of small-pox had been started from the use of virus originated *de novo* by inoculating a cow with small-pox matter.

Dr. Lyman thought that would confirm the received theory, for a first culture would attenuate the disease only to some degree, and inoculation should have been continued in a series of cows.

Dr. Bridge believed that the first inoculations with vaccine virus had been as mild as later ones; while in epidemics, small-pox began severely in every new center.

Dr. Lyman said that epidemics generally developed in communities which had not seen the disease for a long period, and that a new culture was most virulent in fresh soil.

Dr. Bridge said every person was a fresh soil for the disease, but Dr. Lyman believed that exposure to the general influence of the epidemic seemed to mitigate their predisposition.

Dr. E. W. Lee partly explained the cessation of epidemics by an increased vigilance on the part of the people who soon became alarmed and established quarantines.

Dr. Bridge wished to know how to reconcile hereditary tolerance with small-pox inoculations so extensively used in the British Isles. These generally had the disease in a mild form, and in reckoning them with other cases of small-pox, would give this low percentage of death quoted by Dr. Lyman.

Dr. W. E. Clark remembered the time when small-pox was inoculated to insure immunity afterward; his parents had submitted to the disease. Not more than three or four per cent. died.

Dr. W. J. Maynard said contagious impetigo was liable to follow vaccination; he had seen a few cases.

Some of the members had observed that disease in a few cases, and others reported a few cases of death from vaccination with virus kept for some time, or carried in the coat pocket. It seemed the virus had been thus altered and acted somewhat like septicæmic matter.

